# Fabella Syndrome as an Uncommon Cause of Posterolateral Knee Pain after Total Knee Arthroplasty: A Case Report and Review of the Literature

**DOI:** 10.1155/2016/4328462

**Published:** 2016-06-21

**Authors:** Eriko Okano, Tomokazu Yoshioka, Takaji Yanai, Sho Kohyama, Akihiro Kanamori, Masashi Yamazaki, Toshikazu Tanaka

**Affiliations:** ^1^Department of Orthopedic Surgery, Faculty of Medicine, University of Tsukuba, 1-1-1 Tennodai, Tsukuba, Ibaraki 305-8575, Japan; ^2^Division of Regenerative Medicine for Musculoskeletal System, Faculty of Medicine, University of Tsukuba, 1-1-1 Tennodai, Tsukuba, Ibaraki 305-8575, Japan; ^3^Department of Orthopaedic Surgery, Kikkoman General Hospital, 100 Miyazaki, Noda, Chiba 278-0005, Japan

## Abstract

The fabella is a sesamoid bone that is located in the lateral head of the gastrocnemius muscle and has been identified on magnetic resonance imaging in 31% of Japanese people. In the present case, a 65-year-old woman experienced posterolateral knee pain, accompanied by a clicking “sound” during active knee flexion, after undergoing total knee arthroplasty for knee osteoarthritis. Eight months of conservative therapy failed to produce an improvement, with progressive osteoarthritic change of the fabella identified on plain radiography. Based on this evidence, a diagnosis of fabella syndrome was made and the patient underwent a fabellectomy. Fabellectomy provided immediate resolution of posterolateral knee pain and the clicking sound with knee flexion, with the patient remaining symptom-free 18 months after fabellectomy and with no limitations in knee function. Fabellectomy eliminated symptoms in all of five case reports that have been previously published and is regarded as an effective first choice for treating fabella syndrome after total knee arthroplasty.

## 1. Introduction

Pain after total knee arthroplasty (TKA) can result from multiple factors [1, 2]. Fabella syndrome has been identified as an uncommon, but relevant, cause of pain post-TKA due to mechanical irritation of the posterolateral tissues of the knee. The symptoms of fabella syndrome are posterolateral pain and a catching sensation (or clicking sound) with knee flexion [3, 4]. We report the case of a patient presenting with fabella syndrome after routine TKA and include a brief review of literature.

## 2. Case Report

A 66-year-old woman was referred to our specialty clinic for assessment of persistent right knee pain, 8 months after undergoing a TKA for knee osteoarthritis. The patient had undergone right cemented TKA, using a LEGION cruciate-retaining total knee system (Smith & Nephew, Memphis, TN, USA), without patella resurfacing. Range of motion, standing, and walking exercises under full weight bearing were started from the first postoperative day. The pain in her right knee developed one week after her TKA and was localized to the popliteal fossa and was associated with catching/clicking with active knee flexion. The patient had undergone successful left TKA at age of 58, and her medical history was otherwise unremarkable. The patient reported her main occupation as being a housewife.

**Figure 1:**
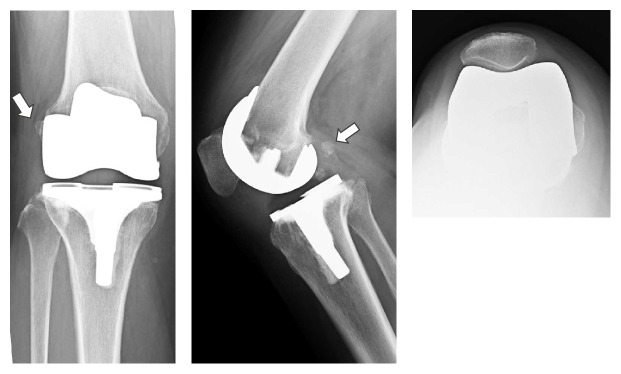
Plain radiographs on first visit; the white arrow identifies the fabella.

**Figure 2:**
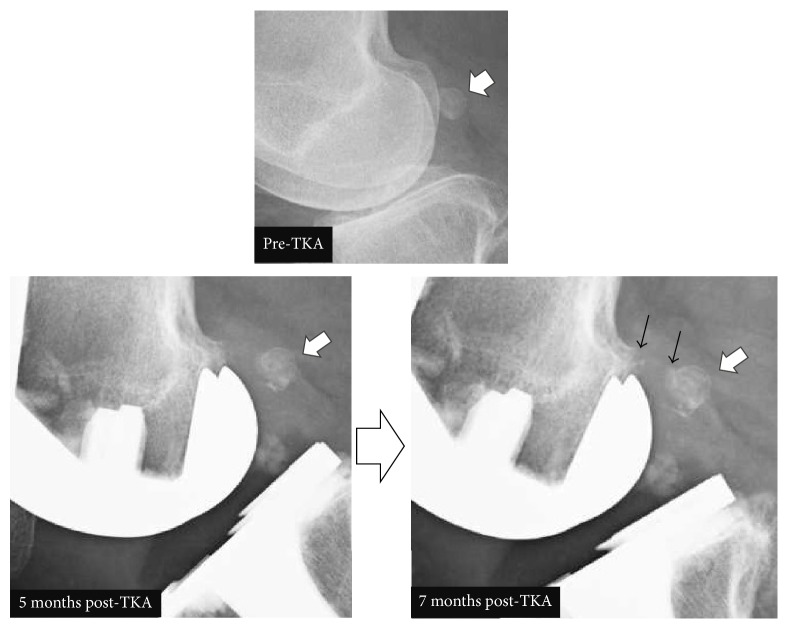
Plain, lateral view radiographs of the knee at several time points; the white arrow identifies the fabella and the black arrow onset and progression of osteoarthrosis.

The physical assessment at the first visit identified the following. Upon visual examination, no redness or swelling was observed, and scarring of the surgical incision, along the midline of the patella, was confirmed. On palpation, no heat or hydrarthrosis was identified. However, deep palpation between the iliotibial tract, on the proximal head of the fibula, and the biceps femoris elicited mild tenderness. A snapping was palpated over the same region during active knee flexion, in the range of 80°–90°, accompanied by pain, identified at an intensity of 91 mm on a 100 mm visual analogue scale (VAS). The snapping was not reproducible on passive knee extension and flexion. Total range of motion of the right knee was 10°–120°, with no obvious instability. Neurological examination and blood work were unremarkable. The Japanese Orthopedic Association (JOA) score was administered. The JOA is as a patient-derived knee scoring system to evaluate physical impairment and disability in patients with knee osteoarthritis that is commonly used in Japanese clinical practice. The JOA score evaluates four domains: pain on walking, pain on ascending or descending stairs, range of motion, and joint effusion [5]. The patient reported a total JOA score of 65, with the following domain-specific scores: pain on walking, 25; pain on ascending or descending stairs, 5; range of motion, 25; and joint effusion, 10. Plain radiographs of the right knee were obtained on the first visit (Figure 1). While standing, the femorotibial angle (FTA) was 174°, and the femoral and tibial component angles were within the normal range: 97° for *α* angle; 88° for *β* angle; 6° for *γ* angle; and 89° for *δ* angle [6]. The posterior condyle offset was 29 mm, with a 1° tibial posterior declining angle [7]. A fabella was identified at the posterior femoral condyle, 11 mm along the major axis of the fabella (Figure 1). Comparison of plain radiographs of the right knee joint at several points in time confirmed presence of a fabella prior to surgery and progressive osteoarthrosis, with osteophyte formation, over the posterior femoral condyle over the 5- to 7-month post-TKA period (Figure 2).

**Figure 3:**
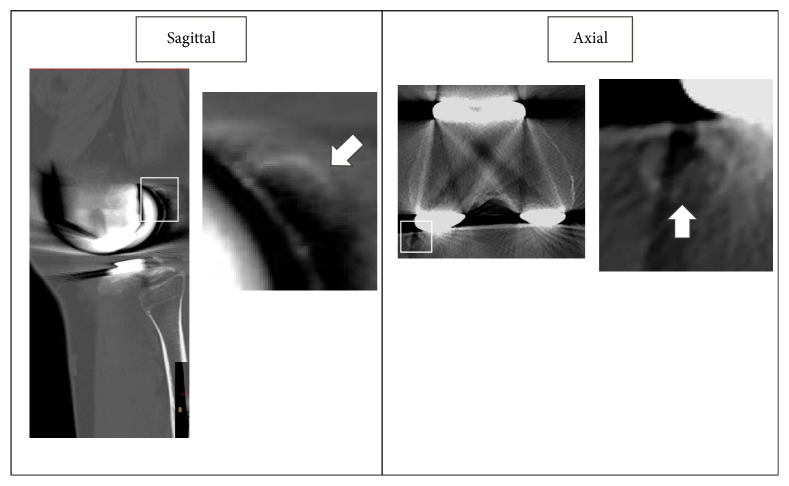
Position of the femoral component and the fabella; the white arrow identifies the fabella.

Review of computed tomography (CT) images obtained at 7 months post-TKA (Figure 3) identified a fabella, 11 × 10 mm in size, over the posterior femoral condyle. The femoral component was fixed in 2° of internal rotation, with respect to the clinical epicondylar axis, and the anterior-posterior axis of the tibial component was aligned with the medial edge of the tibial tuberosity and the PCL attachment. Therefore, there were no abnormal findings in the position of the femoral or tibial components.

Considering that (1) the symptom started one week after TKA surgery, (2) the pain was localized in the posterolateral aspect of the knee, and (3) the pain and snapping were caused by active knee flexion, fabella syndrome was identified as the primary diagnosis. The following were considered as differential diagnoses: (1) impairment associated with remaining cement or resected bone fragments; (2) soft tissue impingement; and (3) popliteal tendinitis.

**Figure 4:**
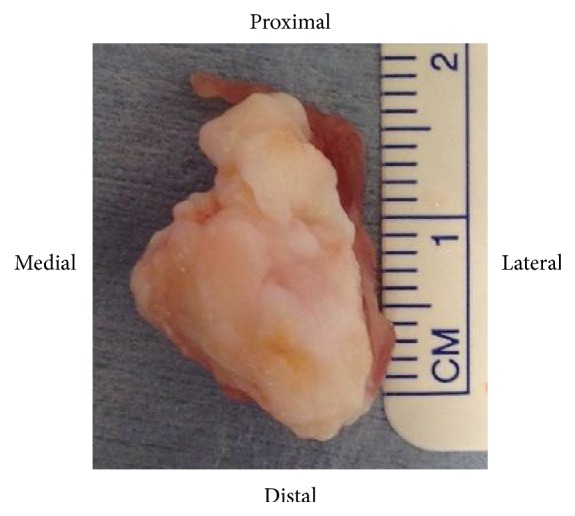
Gross pathology of the excised fabella, confirming significant osteoarthrosis.

Based on our information, we determined excision of the fabella (fabellectomy), through a separate posterolateral incision, to be the treatment of choice. We first performed an arthroscopic examination to diagnostically rule out the potential differential diagnoses. Arthroscopic examination confirmed absence of damage or abrasion to the polyethylene components, with unremarkable findings on visualization of the remaining cement or bone fragments, the popliteal tendon and posterior cruciate ligament, and absence of soft tissue impingement. The extirpation of the fabella was performed using a posterolateral approach between the iliotibial tract and the biceps femoris, confirming the presence of snapping between the femoral component and fabella during knee flexion. The size of the fabella was 20 mm along the major axis, with osteophyte and deformation of the articular cartilage confirmed (Figure 4).

Subjective symptoms resolved immediately after the surgery, and, at 1 month after surgery, range of motion was 0°–120° and the VAS score improved to 10 mm. At 18 months after the surgery, the JOA score had improved to a total score of 85: pain on walking, 30; pain on ascending or descending stairs, 20; range of motion, 25; and joint effusion, 10. There was no recurrence of snapping or pain over the posterolateral aspect of the knee.

## 3. Discussion

Sources of knee pain after TKA can be divided into 4 categories: originating from mechanical or biological factors, with location of the lesion either inside or outside of the knee joint [2]. We based our primary diagnosis of fabella syndrome on the following information: radiographs confirming absence of abnormalities in the positioning of the artificial components; appearance of symptoms 1 week post-TKA; pain localized to the posterolateral area of the knee, specific to the location of the fabella; and a chief complaint of snapping and pain with active knee flexion, which is specific of fabella syndrome.

A summary of the causes and presentation of fabella symptom identified in our review of the literature is provided in Table 1. The major symptoms of fabella syndrome are pain due to a mechanical irritation of local soft tissues during knee extension, causing tension, by pressure from the fabella, on the lateral femoral condyle, regardless of the history of knee surgery [4, 8]. There are five case reports of fabella syndrome that have been documented post-TKA [3, 9–12]. The average age of the patients presenting with fabella syndrome, 1 man and 5 women, was 63, and the time to onset of symptoms after surgery varied between 6 days and 1 year, with an early postoperative occurrence being more common. The major symptoms resulted from mechanical irritation and stress symptoms, such as pain, catching, clicking, and crepitus, in the posterolateral region of the knee with active knee flexion and extension. Two mechanical causes have been reported in the literature, 2 cases resulting from “catching” of the fabella on the polyethylene insert [3, 10], and 3 cases resulting from the fabella being pressed onto the lateral femoral condyle [9, 11, 12]. With regard to fabella syndrome appearing after TKA, the following characteristics have been reported: (1) size of the fabella was ≥1.0 cm along its major axis [3], (2) anatomically exceptional fabella in the lateral head of the gastrocnemius, (3) abnormal placement or mismatch of prosthetic components resulting in impingement of the fabella, and (4) ligament instability possibly caused by an unbalanced distribution of soft tissue [12]. Fabellectomy was the treatment of choice in all reported cases, with immediate resolution of symptoms. Therefore, we believe fabellectomy should be the first choice of treatment in the absence of abnormal positioning and/or size of the TKA components.

In conclusion, we reported the case of one patient presenting with fabella syndrome after TKA. Fabella syndrome should be included as one of the differential diagnoses of post-TKA knee pain, with fabellectomy providing an optimal treatment option.

## Figures and Tables

**Table 1:** Previous reports on fabella syndrome after TKA.

Previous report (published year)	Age/sex M: male F: female	Onset time after TKA	Symptoms	Movement that causes symptoms	Etiology	Treatment
Jaffe et al. 1988 [3]	63 F	6 days	Pain located in the posterolateral part of the knee & snapping & clicking	Knee flexion at 90°	The posterior edge of the polyethylene component	Fabellectomy

Laird 1991 [9]	68 M	3 months	Pain behind the knee & hard lump	During knee flexion & extension	Posterior condyle of the prosthesis	Fabellectomy

Larson and Becker 1993 [10]	67 F	3 months	Pain localized to the posterolateral aspect of the knee & catching	During knee motion	Moderate mediolateral laxity & polyethylene insert	Changed to a thicker polyethylene & fabellectomy

Erichsen 1997 [11]	64 F	1 year	Pain in the lateral part of popliteal fossa & clicking	When the knee was extended from full flexion	The edge of the femoral component	Fabellectomy

Segal et al. 2004 [12]	53 F	8 weeks	Pain at the posterolateral aspect of the popliteal fossa & crepitus	Knee flexion & extension	Lateral edge of the prosthetic femoral condyle	Fabellectomy

Present study	66 F	1 week	Posterolateral knee pain & snapping	Active knee flexion at 80–90°	Lateral edge of the femoral component and femoral condyle	Fabellectomy
